# A Comparison of Two Insulated Electrocautery Blades: What is the Thermal Damage Effect on Transvenous Cardiac Device Leads?

**DOI:** 10.19102/icrm.2018.091206

**Published:** 2018-12-15

**Authors:** Robert D. Schaller

**Affiliations:** ^1^Perelman School of Medicine, University of Pennsylvania, Philadelphia, PA, USA

**Keywords:** Cardiac implantable electronic device, electrocautery, lead management

## Introduction

Electrocautery—referring to a process during which a direct or alternating current is passed through an electrode, generating heat—is used to achieve hemostasis or targeted tissue destruction during surgery. However, it is known that associated adverse consequences such as electromagnetic interference^1,2^ and physical damage to implanted devices^3^ may occur with this technique. As such, newer electrocautery devices are being introduced in an attempt to minimize such collateral damage.

A comparison study, “Damage to Transvenous Leads During Electrocautery—A Comparison of Two Insulated Electrocautery Blades,”^4^ was designed to evaluate the PhotonBlade™ (Invuity, San Francisco, CA, USA) versus the PlasmaBlade™ (Medtronic, Minneapolis, MN, USA) with respect to thermal damage occurring as a result of electrocautery on transvenous cardiac implantable electronic device leads.

We spoke with Dr. Robert Schaller, an investigator of the study, who provided his expert perspectives surrounding major findings and clinical implications of the published results.

## About Dr. Schaller

Robert D. Schaller, DO, MS, FHRS is a clinical cardiac electrophysiologist and Assistant Professor of Clinical Medicine at the Perelman School of Medicine, University of Pennsylvania, in Philadelphia, PA. His research interests include lead extraction and complex ablation techniques, with a clinical interest in atrial fibrillation and ventricular tachycardia ablation and complex lead management. He is a member of the Heart Rhythm Society.

## Interview

***Question:*** What was your involvement in the lead insulation study presented earlier this year at Heart Rhythm 2018 in Boston, MA?

***Dr. Schaller:*** I was part of the investigational team and helped to design and conduct the study as well as report the results. Along with three other physicians from the Hospital of the University of Pennsylvania and Northwestern University, we independently treated all of the leads with both of the systems under investigation.

***Question:*** In your opinion, how does this study compare to any previous work done regarding lead insulation?

***Dr. Schaller:*** Previous studies have evaluated the effects of traditional electrocautery on lead insulation and have shown that all leads are susceptible, with polyurethane and copolymer leads being uniquely vulnerable.^5^ Kypta et al. showed in a retrospective manner that the PlasmaBlade™ is superior to traditional electrocautery and/or scissor dissection in regard to not only lead damage but also to procedural times, hospital length of stay, and hospitalization costs.^6^ Weisberg et al. compared the PlasmaBlade™ to traditional electrocautery in a prospective in vitro study and showed that, once again, polyurethane and copolymer leads sustained the most damage, with more harm seen using the CUT rather than COAG functionality.^7^ The latter represented a finding that did not match our clinical experience. These researchers also showed that utilizing the blade at any setting in a perpendicular direction rather than a parallel direction led to more insulation damage, which is a finding that has been reproduced. The PhotonBlade™ is an alternative insulated cautery blade and our work represents the only study to date that compared these two proprietary systems. This study was performed prospectively in two phases in an effort to directly compare the effects of the PlasmaBlade™ versus the PhotonBlade™, considering both the CUT and COAG modes, as well as by evaluating the systems in perpendicular and parallel orientations while also testing a larger number of leads than that studied in any previous investigation.

***Question:*** What were the overall findings of the study?

***Dr. Schaller:*** Significantly more leads were damaged using the PlasmaBlade™ than the PhotonBlade™—specifically, 75% versus 40%—and higher power resulted in greater mean damage scores. At the commonly used and equivalent settings of PhotonBlade™ CUT 20 W and PlasmaBlade™ CUT 6 W, damage occurred in 13% of treatments with PhotonBlade™ versus 39% using PlasmaBlade™. Interestingly, significantly more leads were damaged in the COAG mode as compared with in the CUT mode (48% versus 2%), a finding that conflicted with the results of previous work. Consistent with prior studies, polyurethane and copolymer leads sustained significantly more damage than did silicone ones, while a perpendicular blade orientation led to more damage than a parallel one.

***Question:*** Did any of the study findings surprise you? If so, why?

***Dr. Schaller:*** The striking difference in lead damage between the two insulated cautery blades tested was noteworthy, as we did not know what to expect when we first were designing this trial. Interestingly, there were no obvious clues during the lead treatment portion of the study that suggested one device would result in significantly more damage versus the other. Additionally, there has been an ongoing debate in the lead management community as to whether the CUT mode or COAG mode is safest when performing cauterization near leads. Although previous work has hinted that the COAG mode is safer, our clinical experience has suggested otherwise. We were pleased to find that CUT was the safer mode in our study and were somewhat surprised by how conspicuous the difference was.

***Question:*** How have the study findings changed (or reinforced) certain protocols or practices in your device implantation cases?

***Dr. Schaller:*** Although I believe that both insulated blades are safe, I prefer to use the PhotonBlade™ when performing cauterization in device pockets with preexisting leads, particularly if I am working around vulnerable insulation due to age or composition. I continue to use the CUT mode when employing a cautery near leads and try to completely avoid the COAG mode if possible when in close proximity to any insulation material.

***Question:*** Do you have any recommendations or lessoned learned that you wish to share with fellow electrophysiologists who are concerned about lead insulation damage?

***Dr. Schaller:*** The data are quite clear that using insulated blades is safer than traditional electrocautery. I would advise anyone to consider employing this technology when working around preexisting leads or if extensive cautery is required during de novo procedures. Our data suggest that the PhotonBlade™ is the safest cautery tool in regard to insulation damage and I would encourage operators to familiarize themselves with this technology. Additionally, consider utilizing the CUT mode when working on leads, particularly if you are in close proximity to vulnerable insulation. It is important to remember that the leads in our study were treated with stationary three-second burns. If using cautery directly on leads in an attempt to dissect them from surrounding tissues, one should try to avoid static application by keeping the blade moving and they should always ensure that the blade orientation is parallel rather than perpendicular.

***Question:*** What other research do you think would be valuable to review if interested in this subject? What future studies do you believe should be conducted on this subject?

***Dr. Schaller:*** There are ample data on the use of scissor dissection, traditional electrocautery, and now insulated cautery blades, both in the cardiology and plastic surgery literature. In regard to the latter, there are unique principles of insulated blades that have important histologic and cosmetic implications that also lend themselves to work in the electrophysiology laboratory. We have tried to encompass all of the relevant studies specific to lead-related procedures in our paper so as to encourage review by the readers. It is also worthwhile to review previous papers concerning the biophysics of unipolar electrocautery, as these concepts are at the core of our electrophysiology procedures. As our study was performed in an in-vitro manner, I feel that future studies should focus on clinical outcomes as they relate to the type of cautery used and the insulation material present. Similarly, as new lead materials emerge and evolve, one must be cautious and cognizant of any consistent clinical observations made when using novel tools and interacting with unique materials.

***Question:*** What has been your personal experience with advanced energy electrosurgery devices?

***Dr. Schaller:*** Lead insulation continues to represent the weakest link in cardiac implantable electronic device (CIED) systems and traditional electrocautery generates temperatures that can pass the melting points of various insulation materials. Because of this, I think that insulated electrocautery devices are now the standard of care for CIED cases with preexisting leads, as they provide a predictable and reproducible experience. From making the initial incision through the skin to freeing up leads from dense scar tissue, an insulated blade has many advantages and almost no clinical downsides. Rarely with insulated blades, the COAG function at the typical settings does not seem to be as effective as traditional electrocautery. This is easily managed by increasing the energy delivered or changing to the spray mode when using the PhotonBlade™.

***Question:*** What aspects of the PhotonBlade™ appeal to you most as a device implanter? How about regarding the Plasmablade™?

***Dr. Schaller:*** The latter has a long track record of safety and efficacy with solid data to support its use over traditional electrocautery. It provides a reproducible experience and allows for extensive cautery use without sacrificing lead safety. The PhotonBlade™ offers a similar experience with the added confidence afforded by the safety results from our study. Additionally, the former offers a proprietary optical illumination system near the tip of the blade that provides clear and uniform light in crevices as well as deep in the pocket. Lastly, it is also compatible with any approved electrosurgical unit including those that power a traditional cautery, providing flexibility in a hospital setting.
